# CNN-MGP: Convolutional Neural Networks for Metagenomics Gene Prediction

**DOI:** 10.1007/s12539-018-0313-4

**Published:** 2018-12-27

**Authors:** Amani Al-Ajlan, Achraf El Allali

**Affiliations:** Computer Science Department, College of Computer and Information Sciences, King Saud University, Riyadh, Saudi Arabia

**Keywords:** Gene prediction, Metagenomics, ORF, Convolutional neural network, Deep learning

## Abstract

Accurate gene prediction in metagenomics fragments is a computationally challenging task due to the short-read length, incomplete, and fragmented nature of the data. Most gene-prediction programs are based on extracting a large number of features and then applying statistical approaches or supervised classification approaches to predict genes. In our study, we introduce a convolutional neural network for metagenomics gene prediction (CNN-MGP) program that predicts genes in metagenomics fragments directly from raw DNA sequences, without the need for manual feature extraction and feature selection stages. CNN-MGP is able to learn the characteristics of coding and non-coding regions and distinguish coding and non-coding open reading frames (ORFs). We train 10 CNN models on 10 mutually exclusive datasets based on pre-defined GC content ranges. We extract ORFs from each fragment; then, the ORFs are encoded numerically and inputted into an appropriate CNN model based on the fragment-GC content. The output from the CNN is the probability that an ORF will encode a gene. Finally, a greedy algorithm is used to select the final gene list. Overall, CNN-MGP is effective and achieves a 91% accuracy on testing dataset. CNN-MGP shows the ability of deep learning to predict genes in metagenomics fragments, and it achieves an accuracy higher than or comparable to state-of-the-art gene-prediction programs that use pre-defined features.

## Introduction

Metagenomics is the analysis of genomes contained in environmental samples, such as soil, seawater, and human gut samples [1–3]. Metagenomics analysis uses modern techniques to study microbial organisms directly in their natural environments, without the need for the isolation and lab cultivation of individual species [4]. Metagenomics has many useful applications in medicine, engineering, agriculture, and ecology [5, 6]. Gene prediction is an important step in the metagenomics pipeline. Gene prediction is the process of finding the location of coding regions in genomics sequences [7, 8]. Early studies identified genes through experiments on living cells and organisms [9], a reliable but expensive task, and current studies use computational approaches to predict genes due to the efficiency of such methods. Computational approaches in gene prediction can be classified as similarity-based and content-based approaches [8, 10]. Similarity-based approaches search for similarities between candidate and existing known genes in public sequence databases. Thus, similarity-based approaches are computationally expensive and miss novel genes. Content-based approaches are a new generation of gene-prediction programs that overcome these limitations. These approaches use various features of sequences, such as codon usage, GC content, and sequence length. They then apply supervised learning or statistical approaches to determine whether a read contains any genes. Metagenomics gene prediction is a challenging task due to short read-length, incomplete, and fragmented nature of the data [7, 11]. Machine learning-based gene prediction programs for metagenomics fragments show promising results [12, 13]. For example, Orphelia [14, 15]and Metagenomics Gene Caller (MGC)[16] use neural networks to predict genes in metagenomics reads, while MetaGUN [17] uses support vector machine (SVM). These gene prediction programs involve feature extraction and feature selection steps. For example, Orphelia uses a two-stage machine learning approach. First, Orphelia extracts some features from each open reading frame (ORF): monocodon usage, dicodon usage, and translation initiation sites (TISs). Then, linear discriminants are used as a dimensionality reduction technique to reduce feature space. Moreover, ORF length and GC content are combined with other features; then, neural networks are used to compute the probability that an ORF encodes a gene. MGC uses the same two-stage machine learning approach, but it creates several training models based on several GC-content ranges to improve the gene prediction task. MGC adds two additional features, monoamino-acid and diamino-acid usage, which improve gene prediction accuracy.

Classical machine learning workflow starts with data cleaning, feature extraction, model learning, and model evaluation. Moreover, classical machine learning algorithms cannot directly process raw data [18]. Representative features are extracted from the raw data, then, feature vectors are supplied into a classifier to obtain an appropriate class. Selection of the significant features that represent the data requires domain knowledge; this step is critical, difficult, and time-consuming, and it can affect the performance of prediction [19, 20]. Computationally, DNA sequences do not have explicit features, and current representations are highly dimensional [21]. In addition, most feature selection methods do not scale well in the case of high dimensionality [19, 22].

Recent approaches in machine learning use deep learning techniques to automatically extract significant features from raw data, such as image intensities or DNA sequences [19, 20, 23, 24]. Deep learning is used widely and successfully in image recognition, speech recognition, natural language processing, computer vision, bioinformatics, and computational biology [18–20]. In the last few years, there has been a growing interest in deep learning approaches due to the availability of large data, computational resources and accurate prediction [21, 23]. In bioinformatics, deep learning approaches are used in functional genomics, image analysis, and medical diagnostics research [21, 23, 25]. Convolutional neural networks (CNNs) are one of the most popular deep neural networks architectures. CNNs automatically detect significant features and eliminate the need for manual feature extraction. Considerable attention has been paid to the application of CNN-based approaches to bioinformatics problems. Collobert et al. [26] first used CNNs for a sequence analysis of generic text. However, few research studies have used CNN-based approaches for biological sequences [25]. These research studies use CNNs trained directly from raw DNA sequences without the use of a feature extraction step [19]. For example, DeepBind [27] uses CNNs to predict the specificities of DNA and RNA-binding sites by discovering new sequence motifs. Gangi et al. [20] use CNNs and recurrent neural networks (RNNs) to identify nucleosomes positioning in sequences. DeepSEA [28] uses CNNs to predict the chromatin effects of sequence alterations with single nucleotide sensitivity. DanQ [29] uses the CNN and RNN frameworks to predict non-coding function directly from sequences. Basset [30] uses CNNs to identify the functional activities of DNA sequences, such as accessibility and protein binding. Meanwhile, CNNProm [24] uses CNNs for prokaryotic and eukaryotic promoter prediction. CNNProm achieves higher accuracy than other promoter prediction programs.

In this paper, we explore the possibility of using a CNN-based approach in gene prediction using metagenomics fragments. The main advantages of using CNNs are simplicity and efficiency, CNNs achieve promising results in various applications.

## Material and Methods

**Table 1:** Testing data

Genomes	GenBank accession no.	GC content (%)
*Archaeoglobus fulgidus*	NC_000917	48.6
*Methanocaldococcus jannaschii*	NC_000909	31.4
*Natronomonas pharaonis*	NC_007426	63.4
*Buchnera aphidicola*	NC_002528	26.3
*Corynebacterium jeikeium*	NC_007164	61.4
*Chlorobaculum tepidum*	NC_002932	56.5
*Helicobacter pylori*	NC_000921	38.9
*Prochlorococcus marinus*	NC_007577	31.2
*Wolbachia endosymbiont*	NC_006833	34.2
*Burkholderia pseudomallei*	NC_006350	67.7
*Pseudomonas aeruginosa*	NC_002516	66.6

The first three genomes are archaea and the remaining are bacterial genomes

### Dataset

We use two datasets, one for training, and the other for testing CNN-MGP. The datasets were used by Orphelia [14] and MGC [16]. The training data included seven million ORFs extracted from 700 bp fragments. These fragments were excised from 131 fully-sequenced prokaryotic genomes (bacterial and archaeal) [14] and their gene annotations obtained from *GenBank* [31]. We divided the training data into 10 mutually exclusive parts based on pre-defined GC ranges. Previous research has shown that building multiple models based on GC content is better than building a single model [16], because fragments with similar GC content have closer features such as codon usage [16]. The testing data included fragments of 700 bp in length from three archaeal and eight bacterial genomes. Table 1 presents the genomes used in the testing, with their *GenBank* accession number and GC content. The 700 bp fragments were randomly excised to create a 1-fold genome coverage from each training genome and a 5-fold coverage for each genome in the testing dataset.

### The Proposed  Method

Our proposed method has three main phases including data pre-processing, training, classification and post-processing. First, we numerically encode the ORFs before inputting them into the CNN models. Then, 10 CNN models are built for the classification phase. Finally, the CNN classifiers are used to approximate the gene probability for the candidate ORFs, and a greedy algorithm is used to select the final gene set.

#### Data Pre-processing

**Fig. 1:**
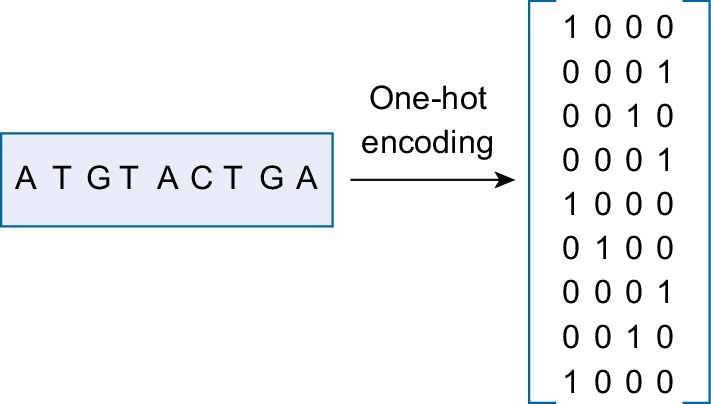
One-hot Encoding for DNA sequence. Each nucleotide is represented as a one-hot vector: A = 1000, T = 0001, C = 0100, and G = 0010

We use character-level one-hot encoding to represent the ORFs similar to previous research [21, 24, 32]. One-hot encoding is used to transform categorical data such as nucleotides into a numerical form. Each nucleotide is represented as a one-hot vector that has all zero entries except one in a specific position. For example, A is encoded as (1,0,0,0), T as (0,0,0,1), C as (0,1,0,0), and G as (0,0,1,0). Each ORF, with length *L*, is represented as *L*$\times$4 matrix (705 is the maximum ORF length in our problem). Figure 1 shows the one-hot encoding for a DNA sequence.

**Fig. 2:**
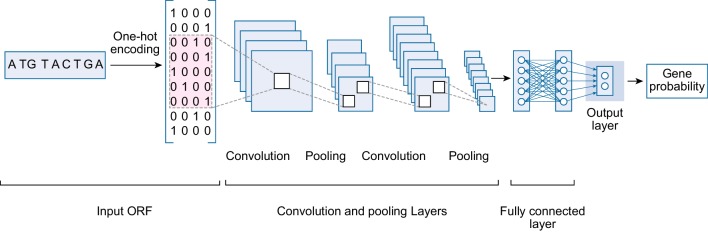
CNN-MGP Architecture. First, an ORF is encoded numerically using one-hot encoding; then, a matrix of numbers is inputted into an appropriate CNN-MGP model based on its fragment GC content. The CNN-MGP model consists of six layers. The first layer is a convolutional layer with 64 filters and a filter window size of 21. The second layer is a max-pooling layer with a pool size of 2. The third layer is a convolutional layer with 200 filters and a filter window size of 21, and the fourth layer is a max-pooling layer with a pool size of 2. Then, the output is flattened to a 1D vector before being inputted into a fully connected layer with 128 neurons. Then, the output layer produces a final gene probability

#### Training

A convolutional neural network (CNN) is a special type of neural networks that works with data having a grid topology [33]. CNNs were developed by LeCun et al. [34] in 1998 to recognize handwritten characters from bank checks. Recently, CNNs have been applied to several applications such as image recognition, video recognition, natural language processing, and computational biology. CNNs are composed of several layers of convolutional, non-linear, pooling, and fully connected layers. The convolutional layer is the most important building block of a CNN. It processes input data using a matrix of weights called a filter, which is a matrix of parameters that are changed by a learning algorithm [33]. Filters, of window size *n*, slide over the input data, and a dot product is calculated between the input data and filter parameters to produce a feature map. The first convolutional layer is able to capture sequence patterns, and deeper convolutional layers can capture patterns that are more complex [35, 36]. After the convolutional layer, a non-linear activation function, the rectified linear unit (ReLU), is applied to the output. Then, the pooling layer is used to reduce the computational cost, memory usage, and number of parameters and to control over-fitting. The max-pooling layer is the most common type of pooling layers. It computes the maximum output from a small window [33], then, a fully connected layer is used to obtain the probability of prediction.

We use one-dimensional CNNs, because DNA sequences are one-dimensional arrays of nucleotides. We use holdout validation to partition data into training and validation sets. In total, 70% of the training dataset is used for training and 30% for validation. The training dataset is used to train models with different hyper-parameters, and the validation set is used to test these models. Hyper-parameters are selected based on the performance of the validation dataset of GC range one. The selection of the number of layers, the number of filters, and filter window size is data- and application-dependent [19, 32]. We follow the testing-based approach used by Zeng et al. [32] and train different models with different configurations to get the most suitable configurations for our problem. First, we use 16 as the number of filters, and we test different filter window sizes: 5, 10, 21, 24, and 30. We find the window size of 21 produce the highest accuracy of 97.71%. Then, we test different number of filters of 16, 32, 64, 128, and 200. The 200 filters produce the highest accuracy of 97.92%. Then, we test two layers with number of filters 64 and 200, which produce the highest accuracy of 98%. Table 2 shows cross-validation of our model with different filter window sizes and number of filters. We select a batch size of 256, which is suitable for most applications. Finally, the model with the best performance, as shown in Fig. 2, is selected to build the final CNN models from the entire training dataset.

We compute the accuracy of CNN-MGP models for each GC range using cross-validation. We use hold-out validation, a type of cross-validation method. The training dataset is divided into two datasets: 70% for training and 30% for validation. Both the training and validation datasets have the same class proportion as the entire dataset. CNN-MGP is trained using a training dataset and is evaluated on validation dataset. Table 3 presents the accuracy of CNN-MGP models, which is between 98 and 99.1%. CNN models with a higher GC range achieve a higher accuracy than those with a lower GC range.

Each model consists of six layers. The first layer is a convolutional layer with 64 filters and a filter window size of 21. The second layer is a max-pooling layer with a pool size of 2. The third layer is a convolutional layer with 200 filters and a filter window size of 21. The fourth layer is a max-pooling layer with a pool size of 2. Then, we use a dropout layer that drops out portions of its output to improve the performance of CNNs and to reduce over-fitting [37]. We set the dropout rate to 50%. Then, the output is flattened to a 1D vector before supplying to a fully connected layer. The fifth layer is a fully connected neural network with 128 neurons. Then, we use a dropout layer. Finally, we use a softmax output layer to estimate the gene probability.

The CNN models are implemented using the Keras package [38], a minimalist Python library for deep learning. It runs on top of TensorFlow [39] and executes on GPUs. We used the Amazon Elastic Compute Cloud (Amazon EC2) to perform our experiments [40].

**Table 2:** The accuracy of the first CNN model with different configurations by varying the number of convolutional layers, the number of filters, and filter window size

No. of convolutional layers	No. of filters	Filter window size	Accuracy
1	16	5	97.57
1	16	10	97.68
1	16	21	97.71
1	16	24	97.70
1	16	30	97.65
1	32	21	97.81
1	64	21	97.87
1	128	21	97.89
1	200	21	97.92
2	(64,200)	21	98.00

**Table 3:** CNN cross-validation performance for different GC ranges

GC range	CNN accuracy
0–36.57	98.0
36.57–41.57	98.4
41.57–46	98.5
46–50.14	98.3
50.14–54.28	98.3
54.28–58.14	98.0
58.14–61.85	98.3
61.85–65	98.8
65–68.28	99.0
68.28–100	99.1

#### Classification and Post-Processing

To predict genes for a given metagenomics fragment, we extract all complete and incomplete ORFs from each fragment. A complete ORF is an ORF that starts with a start codon (ATG, CTG, GTG or TTG) followed by a number of codons and ends with a stop codon (TAG, TAA, or TGA). Incomplete ORF does not have start or stop codons or both. The ORFs are then numerically encoded using one-hot encoding approach. Then, we select an appropriate CNN model to score each ORF based on the GC content of the fragment. The output from the CNN is the probability that an ORF encodes a gene. ORFs with a probability greater than 0.5 are considered as candidate genes. Some of the candidate genes may overlap and only one can be the candidate gene. Genes in prokaryotes can maximally overlap by 45 bp [41]. Therefore, a greedy algorithm [14, 16] is used as a post-processing step to eliminate any overlapping genes and generate a final list of candidate genes. The candidate gene with the highest probability is more likely to be the correct gene, and we remove all candidate ORFs that overlap with it by more than 60 bp.

## Results and Discussion

### Performance Measures

To measure the gene prediction performance, a comparison is made between the algorithm’s predictions and the true gene annotation in the fragments derived from *GenBank* [31]. When the ORF overlaps with at least 60 bp of an annotated gene in the same reading frame it is considered a true positive (TP). On the other hand, if the predicted ORF is incorrectly identified as a gene, it is considered a false positive (FP). Moreover, a false negative (FN) is counted when an overlooked gene is incorrectly identified as a non-coding ORF. We measure the prediction performance based on the sensitivity, specificity, and harmonic mean. Sensitivity is used to measure the probability of detection, as it measures the percentage of genes that are correctly detected. Meanwhile, specificity is used to measure the reliability of the prediction, as it measures the percentage of predicted genes that are annotated. For comparison with the Orphelia and the MGC gene prediction programs, we use the positive likelihood score as a measure of specificity. The sensitivity, specificity, and harmonic mean are computed using the following equations:

1 $$\begin{aligned} \mathrm{Sensitivity}= & {} \frac{\mathrm{TP}_\mathrm{gene}}{\mathrm{TP}_\mathrm{gene}+\mathrm{FN}_\mathrm{gene}} \end{aligned}$$

2 $$\begin{aligned} \mathrm{Specificity}= & {} \frac{\mathrm{TP}_\mathrm{gene}}{\mathrm{TP}_\mathrm{gene}+FP_\mathrm{gene}} \end{aligned}$$

3 $$\begin{aligned} \mathrm{Harmonic Mean}= & {} \frac{2 \times \mathrm{Sens} \times \mathrm{Spec}}{\mathrm{Sens} + \mathrm{Spec}}. \end{aligned}$$

### Results

We evaluate CNN-MGP models on an external dataset. The testing dataset contains fragments of 700 bp in length from three archaeal and eight bacterial genomes, as shown in Table 1. We compare CNN-MGP prediction with true gene annotation from *GenBank* [31]. Moreover, we repeat the testing 10 times per genome. We compute the mean and standard deviation for the sensitivity, specificity and harmonic mean of 10 random replications per genome, as presented in Table 4. CNN-MGP achieves an average specificity of 94.87%, an average sensitivity of 88.27%, and an average harmonic mean of 91.36%. The average standard deviation of the harmonic mean is 0.14%.

We compare CNN-MGP with three state-of-the-art gene prediction programs—Orphelia [14], MGC [16], and Prodigal [42]—using the same test dataset. The results from the comparison are presented in Table 4. CNN-MGP achieves specificity similar to Prodigal, but Prodigal outperforms CNN-MGP in terms of sensitivity and harmonic mean. Prodigal, CNN-MGP, and MGC all outperform Orphelia. CNN-MGP outperforms Orphelia by an average harmonic mean of 10%; its overall performance is similar to that of MGC, with both methods achieving an average harmonic mean of 91% for some genomes, CNN-MGP performs better, while MGC performs better for others.

**Table 4:** Comparison of CNN-MGP, Orphelia, MGC, and Prodigal on testing data

Genomes	CNN-MGP			Orphelia			MGC			Prodigal		
	Sp	Sn	HM	Sp	Sn	HM	Sp	Sn	H.M	Sp	Sn	HM
*A. fulgidus*	94.95$\pm {0.21}$	86.15$\pm {0.19}$	90.33$\pm {0.16}$	88.57$\pm {0.21}$	80.58$\pm {0.17}$	84.38$\pm {0.16}$	95.04$\pm {0.14}$	84.13$\pm {0.23}$	89.31$\pm {0.15}$	95.79$\pm {0.15}$	96.13$\pm {0.08}$	95.96$\pm {0.10}$
*M. jannaschii*	96.13$\pm {0.15}$	93.60$\pm {0.17}$	94.85 $\pm {0.16}$	95.20$\pm {0.17}$	90.46$\pm {0.16}$	92.77$\pm {0.14}$	97.19$\pm {0.12}$	92.63$\pm {0.19}$	94.85$\pm {0.13}$	95.14$\pm {0.14}$	95.15$\pm {0.15}$	95.15$\pm {0.12}$
*N. pharaonis*	96.17$\pm {0.12}$	82.99 $\pm {0.28}$	89.09$\pm {0.18}$	75.99$\pm {0.34}$	68.74$\pm {0.34}$	72.17$\pm {0.33}$	95.28$\pm {0.12}$	85.79$\pm {0.20}$	90.29 $\pm {0.14}$	97.48$\pm {0.10}$	95.77$\pm {0.18}$	96.62$\pm {0.12}$
*B. aphidicola*	97.03$\pm {0.20}$	92.67$\pm {0.41}$	94.80 $\pm {0.26}$	95.54$\pm {0.28}$	89.40$\pm {0.33}$	92.37$\pm {0.22}$	98.01$\pm {0.19}$	91.11$\pm {0.37}$	94.43$\pm {0.23}$	96.65$\pm {0.27}$	96.97$\pm {0.26}$	96.81$\pm {0.25}$
*C. jeikeium*	95.72$\pm {0.11}$	87.37$\pm {0.15}$	91.35 $\pm {0.09}$	79.52$\pm {0.22}$	74.23$\pm {0.23}$	76.79$\pm {0.22}$	96.13$\pm {0.11}$	87.70$\pm {0.23}$	91.72 $\pm {0.17}$	95.31$\pm {0.19}$	94.99$\pm {0.10}$	95.15$\pm {0.10}$
*C. tepidum*	94.46$\pm {0.14}$	81.09 $\pm {0.28}$	87.24$\pm {0.10}$	77.51$\pm {0.22}$	66.95$\pm {0.23}$	71.85$\pm {0.21}$	93.42$\pm {0.14}$	79.08$\pm {0.24}$	85.65$\pm {0.18}$	94.35$\pm {0.14}$	88.15$\pm {0.19}$	91.14$\pm {0.11}$
*H. pylori*	96.24$\pm {0.15}$	91.22 $\pm {0.13}$	93.66$\pm {0.11}$	94.17$\pm {0.20}$	88.99$\pm {0.22}$	91.5$\pm {0.20}$	97.77$\pm {0.14}$	89.70$\pm {0.22}$	93.56$\pm {0.17}$	95.29$\pm {0.14}$	93.07$\pm {0.14}$	94.16$\pm {0.12}$
*P. marinus*	98.15$\pm {0.07}$	89.12$\pm {0.13}$	93.42$\pm {0.07}$	94.41$\pm {0.20}$	84.98$\pm {0.24}$	89.45$\pm {0.20}$	97.71$\pm {0.11}$	87.92$\pm {0.20}$	92.55$\pm {0.12}$	97.52$\pm {0.17}$	91.96$\pm {0.20}$	94.66$\pm {0.15}$
*W. endosymbiont*	82.71$\pm {0.38}$	90.90$\pm {0.27}$	86.61$\pm {0.27}$	86.24$\pm {0.20}$	83.79$\pm {0.20}$	84.99$\pm {0.20}$	88.25$\pm {0.20}$	87.85$\pm {0.20}$	88.05$\pm {0.20}$	81.52$\pm {0.41}$	92.27$\pm {0.25}$	86.56$\pm {0.31}$
*B. pseudomallei*	95.31 $\pm {0.06}$	86.99$\pm {0.12}$	90.96$\pm {0.08}$	69.54$\pm {0.31}$	64.79$\pm {0.22}$	67.08$\pm {0.26}$	94.79$\pm {0.13}$	87.84$\pm {0.25}$	91.18$\pm {0.18}$	94.28$\pm {0.09}$	96.47$\pm {0.09}$	95.37$\pm {0.08}$
*P. aeruginosa*	96.73$\pm {0.08}$	88.86$\pm {0.13}$	92.63$\pm {0.09}$	71.21$\pm {0.20}$	68.40$\pm {0.18}$	69.78$\pm {0.19}$	96.16$\pm {0.09}$	91.70$\pm {0.11}$	93.88$\pm {0.08}$	96.47$\pm {0.05}$	97.88$\pm {0.06}$	97.17$\pm {0.05}$
Average	94.87	88.27	91.36	84.35	78.30	81.19	95.43	87.76	91.40	94.53	94.44	94.43
Average SD	0.15	0.21	0.14	0.25	0.24	0.22	0.15	0.22	0.16	0.17	0.15	0.14

Performance is measured according to the average specificity, sensitivity, and harmonic mean of 10 replications per genome

### Discussion

The aim of our study is to explore the feasibility of using deep learning in metagenomics gene prediction. The results provide important insights into using deep learning for gene prediction, particularly that it is accurate and simple to implement. Feature extraction and feature selection are important steps in most gene prediction programs, as extracting few or irrelevant features reduces the prediction performance [43]. However, extracting a large number of features is computationally expensive and may cause over-fitting. For example, Orphelia and MGC extract thousands of features, such as codon usages, TIS scores, GC content, and ORF lengths. Then, linear discriminants are used to reduce feature space. Further, neural networks are used to predict genes in metagenomics fragments. CNN-MGP is a CNN-based metagenomics gene prediction program that starts with raw ORFs and then applies pre-processing of one-hot encoding to produce a matrix of numbers that will be inputted into CNNs, as presented in Fig. 2. CNN-MGP learns features from the raw data itself and produces the probability that an ORF encodes a gene. CNN-MGP requires fewer steps than MGC and Orphelia. The main advantage of CNNs is their ability to learn features automatically from the raw data itself without the need to define and compute features that require expert knowledge [19, 44]. CNNs perform two main tasks: feature extraction and classification. The convolutional and pooling layers extract significant features automatically, and then a fully connected layer is used to generate the probability of prediction.

Use of CNNs has some limitations. First, training CNNs is computationally expensive, but using efficient computing environments, such as GPUs, can overcome this limitation; most deep-learning frameworks, such as Caffe2, PyTorch, and TensorFlow, support GPU execution to accelerate training. Second, CNNs are prone to overfitting due to large numbers of hyper-parameters that must be tuned, like number of layers, number of filters, filter window size, and type of activation function. There are various solutions to overfitting, including early stopping and dropout; moreover, designing a CNN model architecture and selecting optimal hyper-parameters are crucial steps in improving prediction performance. We test various CNN configurations by changing the number of filters, the filter window size, and the number of layers to obtain the final model. We found that adding more filters and more layers increases the performance of our model, but it also increases the time complexity. Moreover, we found that in a convolutional layer, a large filter window is better than a small window to capture the characteristics of coding and non-coding regions. These results are consistent with previous CNN-based approach studies for biological sequences that suggested a large filter window to predict promoter and DNA binding sites. For example, CNNProm [24] uses a filter window size of 21 and DeepBind [27] uses a filter window size of 24.

Furthermore, a relationship between GC content and prediction accuracy can be observed in Table 3. CNN models with a higher GC range achieve a higher accuracy than those with a low GC range. For example, CNN models built from sequences with GC content greater than 65% achieved higher accuracy than those built from other GC ranges. This finding further supports our hypothesis that fragments with similar GC content have closer features and thus different classification models should be built for different GC contents.

## Conclusion

Recently, considerable attention has been paid to the application of deep learning to various bioinformatics problems. The purpose of the current study is to use CNNs to predict genes in metagenomics fragments and to investigate the effect of CNNs on gene prediction. CNNs have been used successfully in various bioinformatics problems, such as DNA binding site and promoter predictions.

We introduce CNN-MGP, a metagenomics gene prediction program based on a CNN approach. CNN-MGP does not require domain knowledge such as gene features, because CNNs are able to extract significant characteristics directly from raw data. ORFs are encoded numerically and supplied into an appropriate CNN-MGP model. The model produces the probability that an ORF will encode a gene. We test different CNN configurations by varying the number of filters, the filter window size, and the number of layers to produce an accurate model. The best hyper-parameters are selected for the final models. A comparison of CNN-MGP with recent state-of-the-art gene prediction programs Orphelia, MGC, and Prodigal shows that CNN-MGP produces promising results. Our approach supports the recent use of CNNs to biological sequence analysis. Traditional classification approaches are not effective when trying to find genes in erroneous sequences. The reason behind this is the fact that models are built using features that rely on the correct reading frame such as codon bias. Therefore, any frame-shift in the input read will result in a different distribution that does not match the trained models. The question is whether CNN-based models will be able to overcome this issue and enable us to identify the correct features when sequence errors are introduced.
